# LMTK1, a Novel Modulator of Endosomal Trafficking in Neurons

**DOI:** 10.3389/fnmol.2020.00112

**Published:** 2020-06-30

**Authors:** Shin-ichi Hisanaga, Ran Wei, Anni Huo, Mineko Tomomura

**Affiliations:** ^1^Department of Biological Sciences, Tokyo Metropolitan University, Minami-Osawa Campus, Hachioji, Japan; ^2^Department of Oral Health Sciences, Meikai University School of Health Sciences, Urayasu, Japan

**Keywords:** endosome, Rab11, LMTK1, Rab GAP, Cdk5, neurite outgrowth, spine, vesicle transport

## Abstract

Neurons extend long processes known as axons and dendrites, through which they communicate with each other. The neuronal circuits formed by the axons and dendrites are the structural basis of higher brain functions. The formation and maintenance of these processes are essential for physiological brain activities. Membrane components, both lipids, and proteins, that are required for process formation are supplied by vesicle transport. Intracellular membrane trafficking is regulated by a family of Rab small GTPases. A group of Rabs regulating endosomal trafficking has been studied mainly in nonpolarized culture cell lines, and little is known about their regulation in polarized neurons with long processes. As shown in our recent study, lemur tail (former tyrosine) kinase 1 (LMTK1), an as yet uncharacterized Ser/Thr kinase associated with Rab11-positive recycling endosomes, modulates the formation of axons, dendrites, and spines in cultured primary neurons. LMTK1 knockdown or knockout (KO) or the expression of a kinase-negative mutant stimulates the transport of endosomal vesicles in neurons, leading to the overgrowth of axons, dendrites, and spines. More recently, we found that LMTK1 regulates TBC1D9B Rab11 GAP and proposed the Cdk5/p35-LMTK1-TBC1D9B-Rab11 pathway as a signaling cascade that regulates endosomal trafficking. Here, we summarize the biochemical, cell biological, and physiological properties of LMTK1.

## Introduction

Neurons are highly polarized cells with two types of processes called the axon and dendrite. The axons that extend from a neuron interact with the dendrites of other neurons and form synapses, through which they communicate with each other. These neuronal circuits are the structural and functional basis of higher brain functions, such as cognition and behaviors. Therefore, proper neural connections must be established to maintain physiological brain functions. The establishment of axons and dendrites is a complicated process involving the cytoskeletal organization and membrane trafficking (Arimura and Kaibuchi, 2007; Sann et al., 2009; Polleux and Snider, 2010; Bloom and Morgan, 2011). When axons and dendrites are growing, the membrane surface expands. Membrane components, both lipids and membrane proteins, are required for membrane expansion. These membrane components are synthesized in the cell body and transported to the growing tips (Ng and Tang, 2008; Yap and Winckler, 2012). A supply of membrane components is also needed in mature neurons for maintaining and remodeling the connections, depending on neuronal activities (Bosch and Hayashi, 2012). However, the mechanism regulating the supply of these components to fulfill the requirements is not completely understood.

The membrane components are transported as a small membrane vesicle in cells. A complicated system regulating vesicle transport exists to deliver the vesicles to the proper places. The major regulators are members of the Rab family of small GTPases (Takai et al., 2001: Zerial and McBride, 2001; Stenmark, 2009). The activity of Rab proteins is determined by GTP or GDP bound to them; a GTP-bound form is active and a GDP-bound form is inactive. The exchange between the GTP- and GDP-forms are regulated by Rab guanine nucleotide exchange factor (Rab-GEFs) and Rab GTPase activating proteins (Rab-GAPs), respectively. Active Rabs interact with downstream effector proteins to elicit biological actions. More than 60 Rab family members have been identified, each of which localizes in particular membrane compartments with a specific function (Fukuda, 2008; Hutagalung and Novick, 2011). To date, most functional studies of Rabs and their regulation of membrane trafficking have been performed using nonpolarized cultured cell lines because of their relative ease of handling and observation. A well-documented transport system is endosomal trafficking and related Rabs. Endocytosed membrane proteins are incorporated into Rab5-dependent early endosomes and sorted to Rab7-dependent late endosomes for degradation, to plasma membranes *via* Rab4-dependent rapid recycling or to the perinuclear endosomal recycling compartment (ERC) for the Rab11-dependent slow recycling (Ullrich et al., 1996; Maxfield and McGraw, 2004). Nevertheless, a comprehensive understanding of this process has not been achieved. In particular, the regulation by Rab species-specific GEFs and GAPs remains to be investigated.

In highly polarized neurons, many Rabs are reported to participate in various neuronal activities (D’Adamo et al., 2014: Shikanai et al., 2018). Newborn neurons migrate from their birthplace to the resident place. Rab5, Rab7, Rab11, and Rab18 are involved in the migration (Shikanai et al., 2018). When neurons extend neurites, vesicles must be targeted to distinct membrane compartments, such as the axon or dendrites over long distances. Many Rabs, including Rab5, Rab6, Rab8, Rab11, Rab17, Rab18, Rab21, Rab33A, and Rab35, are reported to work in the neurite outgrowth (Seabra et al., 2002; Nakazawa et al., 2012; Numrich and Ungermann, 2014; Hausott and Klimaschewski, 2016; Villarroel-Campos et al., 2016; Park, 2018). Specifically, in neurons, there is local recycling of vesicles. For examples, the recycling of neurotransmitters within the presynaptic region is regulated by Rab3, Rab4, Rab5, Rab11, Rab27 and Rab35 (Binotti et al., 2016), and that of neurotransmitter receptors within dendritic spines are controlled by Rab4, Rab5, and Rab11 (Kiral et al., 2018). Moreover, mutations of Rabs or Rab-related proteins are known for developmental and degenerative disorders; those are Rab7 in Charcot-Marie-Tooth disease type 2B (CMT2B); Rab3GAP in Warburg Micro Syndrome, Rab27A in Griscelli syndrome type 2, Rab39B in X-linked mental retardation, and LARK2 in Parkinson’s disease (Kiral et al., 2018; Shikanai et al., 2018; Kuwahara and Iwatsubo, 2020). Rab7 and Rab11 are implicated in neurodegenerative diseases such as Alzheimer’s disease (AD) and Huntington’s disease (Kelly et al., 2012; D’Adamo et al., 2014; Kiral et al., 2018). However, much less is known about the regulation mechanism of these Rabs particularly related to neuron-specific activities (for more details on Rab and Rab-related proteins in neurons, see other articles in this special topic on “Membrane Trafficking in Neuron Differentiation and Polarization”).

**Figure 1:**
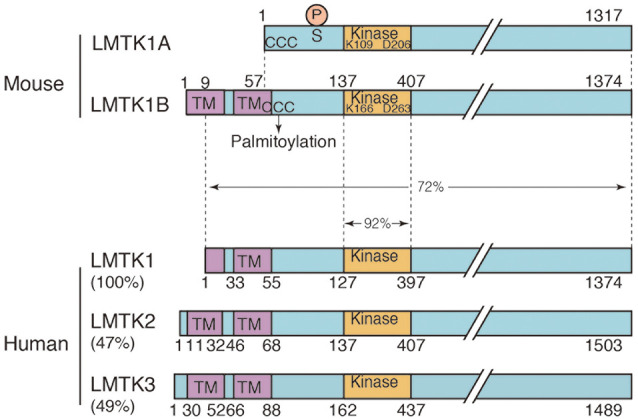
Domain structure of the two isoforms of mouse lemur tail (former tyrosine) kinase 1 (LMTK1) and human LMTK1-3. Mouse LMTK1A and LMTK1B are alternatively spliced isoforms of LMTK1 that consist of 1,317 and 1,374 amino acids, respectively. The kinase domain (Kinase) is present in the N-terminal region with the long C-terminal tail. The amino acids Lys (K) and Asp (D) that are essential for the kinase activity are conserved. LMTK1A is a cytosolic protein that binds to the cytoplasmic surface of endosomal membranes *via* palmitoylation at N-terminal three cysteines (ccc). LMTK1B is a transmembrane protein with two transmembrane sequences at the N-terminus, which is also suggested to be palmitoylated at cysteines (ccc). The Cdk5 phosphorylation site is Ser34 of LMTK1A. Human LMTK1-3 is shown below the mouse LMTK1. Human LMTK1 is translated from the methionine in the first transmembrane sequence according to the amino acid sequences (NCBI accession number: NP_001073864.2). While the identity of the kinase domain is 92%, the identity of the full-length protein is 73% between mouse and human LMTK1, which is a relatively low value. Human LMTK2 (NCBI accession number: NP_055731.2) and LMTK3 (NCBI accession number: NP_001073903.1) are also shown below with % homology to human LMTK1.

LMTK1, initially called AATYK, is an uncharacterized Ser/Thr kinase that is abundantly expressed in the brain (Baker et al., 2001; Tomomura et al., 2001). AATYK was originally isolated as a gene that was upregulated in myeloid precursor cells undergoing apoptosis and named Apoptosis-Associated Tyrosine Kinase (AATYK) because of the amino acid sequence homology in the kinase domain to receptor tyrosine kinases (Gaozza et al., 1997). However, it was later shown to be abundantly expressed in neurons (Baker et al., 2001; Tomomura et al., 2001) and renamed LeMur Tyrosine Kinase 1 (LMTK1) after the long C-terminal tail (see Figure 1; Manning et al., 2002). LMTK1 is now considered a Ser/Thr kinase based on the Ser/Thr kinase activity of its family member LMTK2. As shown in our recent study, LMTK1 regulates axon outgrowth, dendrite arborization, and spine formation through Rab11-dependent vesicle transport. Here, we summarize our knowledge of LMTK1 and its regulation of Rab11 and Rab11-dependent membrane trafficking in neurons.

In the recent reviews of LMTK2 (Bencze et al., 2018) and LMTK (Wendler, 2018), “Lemur Tail kinase” is proposed as the alternative name for LMTK. According to the proposal, we will designate LeMur Tail Kinase 1 as LMTK1 hereafter.

## Gene, Transcripts, and Expression of LMTK1 Isoforms

*Aatk* is the name of the gene encoding LMTK1. The human *aatk* gene is located on chromosome 17q25.3 and the mouse gene is located on chromosome 11; 11 E2. A Northern blot analysis reveals the highest expression of the transcript in the brain, with low levels in most peripheral tissues, such as the heart, spleen, lung, liver, skeletal muscle, kidney and testis (Gaozza et al., 1997; Tomomura et al., 2001). The LMTK1 mRNA is detected in most parts of the mouse brain, including the cerebral cortex, hippocampus, and cerebellum (Baker et al., 2001; Tomomura et al., 2001). *In situ* hybridization revealed the expression of the LMTK1 mRNA in neurons and some non-neuronal cells in the white matter (Tomomura et al., 2001). The latter may be oligodendrocytes, consistent with a previous report (Jiang et al., 2018). The expression of the LMTK1 mRNA increases with neuronal differentiation, as its expression is undetectable in undifferentiated neurons and the highest expression is observed in mature neurons (Tomomura et al., 2003; Wei et al., 2020). However, the regulation of gene expression is not known yet.

Both the mouse and human *aatk* genes contain 15 exons, and multiple transcripts have been reported (Gaozza et al., 1997). The mRNAs are detected as a 5.1 and 5.5 kb doublet in several tissues (Gaozza et al., 1997), or mainly as a 5 kb transcript in mouse brains and an additional small amount of a 5.5 kb transcript in the hippocampus and midbrain (Tomomura et al., 2001). Smaller transcripts of 2.0 and 2.5 kb are detected in the liver and, to a much lesser extent, the skeletal muscle. Thus, LMTK1 is suggested to exist in several alternatively spliced forms in a tissue-specific manner.

At least two major isoforms of LMTK1 are expressed in the mouse brain, LMTK1A and LMTK1B, which differ in the presence or absence of the N-terminal transmembrane sequences (Figure 1; Tomomura et al., 2007; Wei et al., 2020). These isoforms are expressed at similar levels in several brain regions and display a similar increase in expression with the development of the cerebral cortex and cerebellum (Wei et al., 2020). On the other hand, only LMTK1B is reported in the human genome (NCBI accession number: NP_001073864.2), which is translated from the Met located in the first transmembrane sequence (Figure 1). The isoforms and expression of human LMTK1 remain to be examined.

Notably, the microRNA miR-338 is generated from an intron of the *aatk* gene (Gokey et al., 2012; Kos et al., 2012). Its expression has been reported in pancreatic β-cells and Schwann cells, and it is upregulated in cancers. Since its expression deregulates the host *aatk* gene, its transcriptional regulation may intricately control the function of LMTK1 protein.

## Biochemical Properties of LMTK1

### The Kinase Activity of LMTK1

LMTK1 was initially suggested to be a tyrosine kinase based on the homology of the amino acid sequence in the kinase domain to receptor tyrosine kinases (Gaozza et al., 1997). However, LMTK1 is now predicted to be a Ser/Thr kinase, based on the kinase activity of LMTK2 (Wang and Brautigan, 2002, 2006; Kawa et al., 2004). The kinase activity of LMTK1 itself has not yet been clearly shown, although autophosphorylation has been reported (Gaozza et al., 1997; Tomomura et al., 2001; Kawa et al., 2004). Nevertheless, the kinase activity appears to be functionally important, based on experiments using the kinase negative (KN) mutant of LMTK1. LMTK1A has essential and conserved amino acids, such as Lys109 and Asn206, in the kinase domain at the ATP-binding and kinase active sites, respectively. Their mutation, particularly the D206V mutant, results in dominant-negative phenotypes (Tomomura and Furuichi, 2005), similar to the knockdown (KD) or knockout (KO) of the *aatk* gene (Takano et al., 2012; Nishino et al., 2019). This activity is affected by the posttranslational modification of LMTK1, thus suggesting that LMTK1 activity is regulated. Detection of the kinase activity and identification of the specific substrate proteins would be a critical issue to improve our understanding of LMTK1 functions and regulation, although it would be challenging because of the weak kinase activity, which may be capable of phosphorylating proteins only in close proximity.

### A Possible Role for LMTK1 as a Scaffold Protein

LMTK1 has a long C-terminal proline-rich tail with 19 PxxP phospho-tyrosine binding motifs. Several proteins are reported to bind to the C-terminal region. The cyclin-dependent kinase 5 (Cdk5) activator p35 binds to LMTK1 at the C-terminal region of the kinase domain (Honma et al., 2003). LMTK2 is also isolated as a p35 Cdk5 binding protein (Kesavapany et al., 2003). LMTK1A binds Ste20-related proline-rich kinase (SPAK; Piechotta et al., 2003) and protein phosphatase 1 (PP1; Gagnon et al., 2007). Gagnon et al. (2007) proposed that LMTK1 regulates the activity of the Na-K-2Cl cotransporter (NKCC1) by recruiting both the NKCC1-phosphorylating kinase SPAK and dephosphorylating enzyme PP1. PP1 was initially shown to bind LMTK2 (Wang and Brautigan, 2002), a property that may be shared by the LMTK family (Yao et al., 2017). Myosin VI is an LMTK2-binding protein (Chibalina et al., 2007; Inoue et al., 2008). Although this interaction has not yet been identified for LMTK1, LMTK1 would likely participate in endosomal transport in the actin filament (AF)-rich cell periphery. We recently showed that LMTK1 binds to TBC1D9B Rab11 GAP (Nishino et al., 2019), as described below.

### Posttranslational Modification of LMTK1

LMTK1 is subject to several posttranslational modifications. LMTK1 is a phosphoprotein. LMTK1A is phosphorylated at Ser34 in the N-terminal region of the kinase domain by Cdk5/p35 (Figure 1; Tsutsumi et al., 2010). The phosphorylation of this site regulates LMTK1 activity. Interestingly, LMTK2 is also phosphorylated by Cdk5/p35 at Ser1418 in the C-terminal region (Manser et al., 2012). The site is dephosphorylated by PP1, which binds to LMTK2. LMTK3 has also possible phosphorylation sites. Based on these results, Cdk5-mediated phosphorylation appears to be a common regulatory mechanism of LMTK family kinases. Additionally, LMTK1A is phosphorylated by a PKCδ and glycogen synthase kinase-3β (GSK-3β) in cerebellar granule neurons undergoing low K^+^ -induced cell death (Tomomura and Furuichi, 2005; Hughes et al., 2010). The phosphorylation sites are not determined definitely, although the phosphorylation is suppressed by mutations of LMTK1A at Ser480, Ser558, and Ser566 (Tomomura and Furuichi, 2005). LMTK1A is further phosphorylated at Tyr25 and Tyr46 in the N-terminal region by the Src non-receptor type tyrosine kinase (Tsutsumi et al., 2008). Tyr phosphorylation is regulated by membrane association and phosphorylation at Ser34 by Cdk5-p35 (Tsutsumi et al., 2010). The significance of these phosphorylation events is not yet known.

LMTK1A is palmitoylated at the three cysteine residues, Cys4, 6, and 7, in the N-terminus (Figure 1). Palmitoylation is essential for LMTK1A to bind to recycling endosomes and localize to the pericentrosomal region (Tsutsumi et al., 2008). LMTK1B may also be palmitoylated at Cys61, 63, and 64 (Figure 1) because mutations at these sites alter the cellular localization (Wei et al., 2020).

## Subcellular Localization and Regulation of Endosomal Trafficking

### LMTK1 Localizes to Rab11-Positive Recycling Endosomes

**Figure 2:**
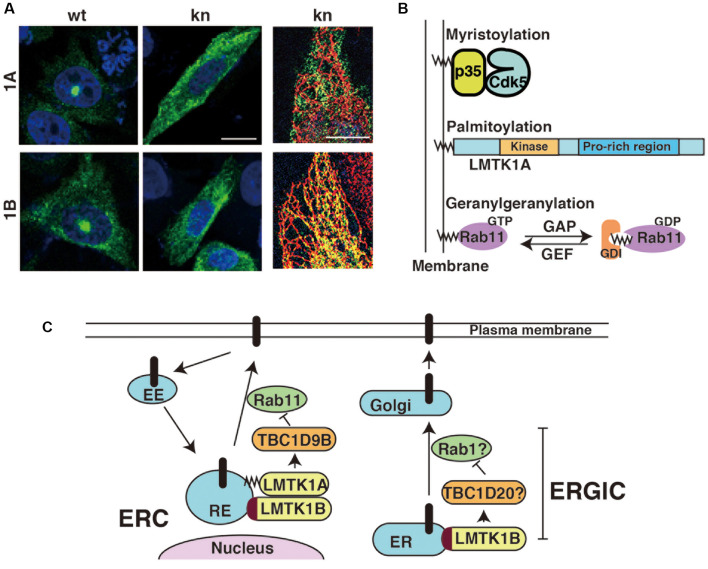
Subcellular localization of LMTK1A and LMTK1B and the regulation of vesicle trafficking at the perinuclear endosomal recycling compartment (ERC) or ERGIC. **(A)** Immunostaining of wild type (WT) and kinase negative (KN) mutants of LMTK1A (1A) and LMTK1B (1B) expressed in CHO-K1 cells. SIM superresolution microscopic images in the right show the localization of LMTK1B KN, but not LMTK1A KN, on ER stained with a KDEL ER marker. Bars represent 20 and 5 μm, respectively (modified from Wei et al., 2020). **(B)** Proteins regulating endosomal trafficking bind to endosomal membranes through different binding modes. Cdk5 associated with the membranes through the myristoylation of the N-terminal glycine of its activator protein p35. LMTK1A accumulates on endosomes through the palmitoylation of cysteine residues in the N-terminal region. Active Rab11 binds to endosomes *via* the geranylgeranylation of the C-terminal cysteine. GDI is a GDP dissociation inhibitor, which binds to the GDP-bound form of Rabs in the cytosol. **(C)** LMTK1A and LMTK1B bind to recycling endosomes (RE) at the perinuclear ERC and regulate Rab11-dependent endosomal transport through TBC1D9B Rab11 GAP. LMTK1B is a transmembrane protein that would be synthesized on the ER and transported to the perinuclear ERC *via* vesicle transport. Because LMTK1B KN is localized to the elongated tubular ER, LMTK1B likely regulates vesicle transport at the ERGIC. The mechanism might be similar to transport at the perinuclear ERC; TBC1D20 and Rab1 are described as possible downstream candidates. EE represents the early endosome and Golgi represents the Golgi apparatus.

LMTK1 is a membrane-bound protein kinase; however, the binding modes of the two isoforms are different. LMTK1A is a peripheral membrane protein tethered on the cytoplasmic surface of the membranes through palmitoylation (Tsutsumi et al., 2008). Although palmitoylation is a reversible lipid modification of proteins (Salaun et al., 2010; Aicart-Ramos et al., 2011), most LMTK1A molecules appear to be palmitoylated, because LMTK1A is recovered in the transferrin receptor-enriched membrane fraction (Tsutsumi et al., 2008; Takano et al., 2010; Nishino et al., 2019). Immunofluorescence staining reveals the most intense localization of LMTK1A to the Rab11-positive recycling endosomes in the perinuclear or pericentrosomal ERC (Figure 2A; Takano et al., 2010, 2012). The kinase activity may be involved in the localization. The D206V KN mutant of LMTK1A is distributed throughout the cytoplasm (Takano et al., 2010).

Interestingly, both upstream and downstream proteins of LMTK1A are associated with endosomal membranes through different types of lipid modifications: Cdk5 binds to membranes *via* the N-terminal myristoylation of its activator protein p35, LMTK1 is palmitoylated at the N-terminal region of transmembrane sequences and Rab11 is modified by geranylgeranylation (Figure 2B). An intriguing question is how those different lipid modifications are coordinately used to assemble these factors on particular endosomes.

LMTK1B is an integral membrane protein with two transmembrane domains at the N-terminus (Figure 1; Tomomura et al., 2007). The first transmembrane domain is composed of 20 amino acids beginning from the first methionine. The second transmembrane domain comprises 21 amino acids and contains a cysteine, one of three possible palmitoylation sites. The palmitoylation of Cys in the transmembrane domain tilts the transmembrane region, affecting the membrane compartments where the palmitoylated proteins are located (Blaskovic et al., 2013). A study has investigated the topology of LMTK2, which also contains two transmembrane domains at the N-terminus. According to the report (Nixon et al., 2013), the C-terminal region faces the cytoplasm. Because LMTK1B shares the same topology, the functional domains, such as the kinase domain and C-terminal proline-rich tail, are all located in the cytoplasm. LMTK1B with the signal sequence should be synthesized on ER-bound ribosomes but localizes to the pericentrosomal ERC (Figure 2A), similar to cytosolic LMTK1A. An interesting question is how both LMTK1B and LMTK1A are targeted to the same pericentrosomal ERC. LMTK1B would travel to the pericentrosomal ERC from the ER through the Golgi apparatus and plasma membranes (Figure 2C). In contrast, LMTK1B KN accumulates in the tubular ER (Figure 2A). If LMTK1B KN functions as a dominant-negative protein, LMTK1B likely participates in the vesicle transport from the ER to the Golgi apparatus, the region designated the ER-Golgi intermediate compartment (ERGIC), probably through an analogous mechanism to LMTK1A in the pericentrosomal ERC. If so, Rab1 and TBC1D20 would be potential downstream targets of LMTK1B (Figure 2C; Haas et al., 2007; Sklan et al., 2007; Ortiz Sandoval and Simmen, 2012; Yang et al., 2016).

Compared to flat cultured cells, in which the localization of exogenously expressed LMTK1 has been examined, the exact membrane compartments of LMTK1 in neurons are not easy to observe and determine. The cytoplasmic area in the soma of neurons is relatively small and it contains large amounts of various membrane organelles (Renvoisé and Blackstone, 2010). Moreover, because of the lack of a reliable antibody for the immunostaining of endogenous LMTK1, the localization of endogenous LMTK1 is not yet known. Exogenously expressed LMTK1A and 1B have been used to study their localization in primary neurons. Both proteins are located in the perinuclear region and display substantial colocalization with Rab11 rather than other endosomal Rabs (Wei et al., 2020). The D206V KN mutant displays a slightly diffuse distribution. LMTK1A is located on Rab11-positive small and moving recycling endosomes in axons and dendrites (Takano et al., 2012).

### LMTK1 Negatively Regulates Endosomal Trafficking in Axons, Dendrites, and Spines

**Figure 3:**
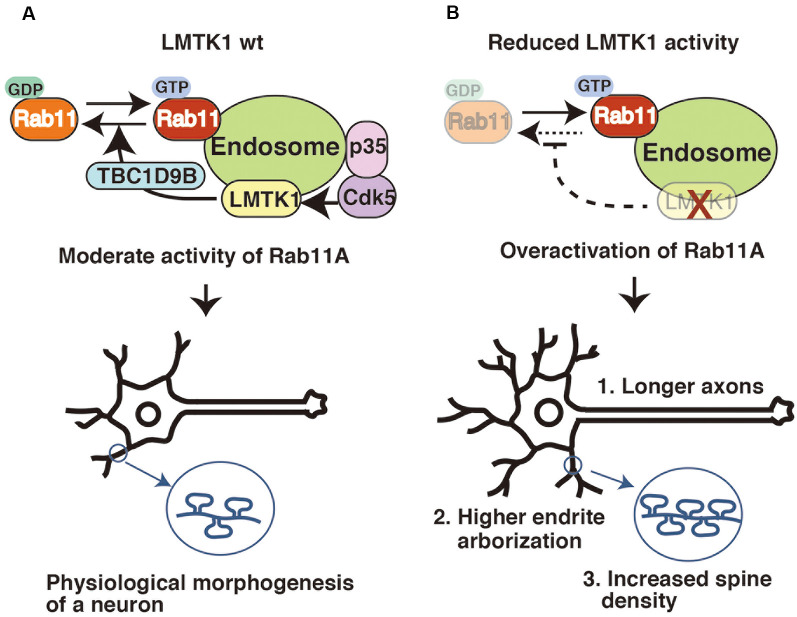
LMTK1 regulates axon outgrowth, dendrite arborization, and spine density through TBC1D9B Rab11 GAP. **(A)** The proper regulation of Rab11A is important for controlling the morphogenesis of neurons. The Cdk5-LMTK1A-TBC1D9B pathway is the upstream cascade of Rab11A endosomal regulation. Cdk5/p35 regulates LMTK1A activity by phosphorylation. LMTK1 activates TBC1D9B, leading to the inactivation of Rab11A activity. This regulation cascade operates on the endosome. **(B)** Downregulation of LMTK1 results in overactivation of Rab11A, leading to: (1) overgrowth of axons; (2) higher dendrite arborization; and (3) excessive dendritic spine formation.

LMTK1 negatively regulates endosomal trafficking in neurons. Although the overexpression of wild type (WT) LMTK1A does not noticeably affect its trafficking, its KO or KD alters various aspects of endosomal movements in neurons. Primary neurons recapitulate their maturation processes in culture, as they first extend an axon, followed by dendrites, and finally form synapses between these structures (Dotti and Banker, 1987; Takano et al., 2015). Surface areas increase with the formation of axons, dendrites, and spines (postsynaptic protrusions of excitatory synapses), and Rab11 contributes to supply the membrane components required for their formation and growth (Park et al., 2004; Brown et al., 2007; Eva et al., 2010; Kawauchi et al., 2010; Yap and Winckler, 2012). LMTK1 KD stimulates all these Rab11-dependent movements, resulting in their overgrowth (Figure 3). The expression of LMTK1A KN also causes the same phenotypes.

Rab11-positive endosomes travel on microtubules (MTs) in the axons and dendrites of primary neurons. These movements of endosomes have been compared between the axons and dendrites, and between WT and LMTK1 KO neurons (Takano et al., 2014). The polarity of MTs differs between these structures; all MTs have the plus end in the distal regions of axons, but the polarity is mixed in dendrites (Baas et al., 1988; Stepanova et al., 2003). The plus end-directed transport is driven by kinesin, and minus-end transport is by cytoplasmic dynein (Hirokawa and Takemura, 2005). Rab11-positive endosomes are transported both anterogradely (plus-end-directed) and retrogradely (minus-end-directed), with a higher percentage of anterograde transport in neurons undergoing axon outgrowth, resulting in the net supply of the membrane components to growing tips. The ratio of the anterograde movement is increased in LMTK1 KO neurons, causing an increased supply and then overgrowth (Takano et al., 2014). Furthermore, the speed and run length of moving endosomes are increased. In dendrites, due to the presence of MTs with both polarities, the bidirectional movements of vesicles are considerably increased compared to axons. In LMTK1 KO neurons, anterograde movement is increased and the retrograde movement is decreased, leading to the oversupply (Takano et al., 2014). Thus, these movements adequately reflect the polarity of MTs in the axons and dendrites and explain the mechanism of overgrowth of the axons and dendrites induced by LMTK1 downregulation.

After the axon has elongated, dendrites begin to grow (Dotti and Banker, 1987; Takano et al., 2015). At that time, neurons would change the transport direction from the axon to the dendrite. The axon often extends from the particular region of the soma in which the perinuclear ERC is located. On the other hand, the dendrites are generally formed on the opposite side of the soma. However, the perinuclear ERC does not change its position but rather alters the direction of the transport to the dendrites, at least in primary cortical neurons (Takano et al., 2014). The exclusion of Rab11 from axons would also contribute to this change (Koseki et al., 2017). In LMTK1 KO neurons, budding of the vesicles from the perinuclear ERC increases, and the moving speed is also enhanced (Takano et al., 2014). These factors might induce the overgrowth of the dendrites.

Growth cones and spines are AF-rich structures that are formed at the distal end of neurites and along dendritic shafts, respectively. The growth cone navigates elongating axons to the target neurons (Tojima and Kamiguchi, 2015), and spines form synapses with presynaptic axon terminal of excitatory neurons (Hutsler and Zhang, 2010; Yuste, 2011; Choquet and Triller, 2013; Spires-Jones and Hyman, 2014). Their proper formation is essential for correct neural connections and communication. Rab11-dependent endosomal transport is implicated in their formation (Park et al., 2006; Eva et al., 2010; Hoogenraad et al., 2010; Esteves da Silva et al., 2015; Bodrikov et al., 2017) and LMTK1 regulates this process (Takano et al., 2014; Nishino et al., 2019). The number of vesicles entering the growth cone and the density of spines is increased in LMTK1 KO neurons (Takano et al., 2014; Nishino et al., 2019). Additionally, the formation of spines is accelerated, rather than a reduction in elimination (Nishino et al., 2019), indicating an oversupply of the membrane components, consistent with other properties of LMTK1 KO neurons.

Endosomal vesicles must approach to and fuse with the plasma membrane to expand the cell surface area. At that time, the transport system would change from the MT-dependent movements to AF-dependent transport. For this conversion, LMTK1 may be inactivated as shown by the fact that while active LMTK1A localizes in the MT-rich neurite shaft, LMTK1A KN localizes in the AF-enriched cell periphery (Sharma et al., 2016). Rab species would also be exchanged, from Rab11 for the MT-dependent movements to another Rab for the AF-dependent transport. A possible Rab is Rab8, although Rab8 is suggested to be involved in whole processes from endocytosis to exocytosis (Peränen, 2011). Recent reports demonstrate the interaction of Rab8 with actin-based motor myosin V and VI (Sahlender et al., 2005; Grigoriev et al., 2011). Further, the relay of vesicles from Rab11 to Rab8 is shown in primary cilia formation (Knödler et al., 2010; Westlake et al., 2011; Chiba et al., 2013), AMPA-receptor transport in spines (Brown et al., 2007) and neurite outgrowth in PC12 cells (Homma and Fukuda, 2016). In the case of ciliogenesis, Rabin8 mediates the relay as an effector of Rab11 and GEF of Rab8 (Westlake et al., 2011; Chiba et al., 2013). During axon elongation, GRAB, which is also an effector of Rab11 and GEF of Rab8, acts as a mediator between two Rabs. GRAB is conveyed on Rab11-positive endosomes in axons as a Cdk5-phosphorylated inactive form (Furusawa et al., 2017) and may be activated at the end of axons. The activated GRAB would recruit and activate Rab8 on Rab11-positive endosomes (Furusawa et al., 2017), converting the transport system to AF-dependent one.

As shown in our recent study, LMTK1 regulates Rab11 activity *via* TBC1D9B Rab11 GAP (Nishino et al., 2019). TBC1D9B is a member of the family of Tre2-Bub2-Cdc16 (TBC) domain-containing proteins (Dabbeekeh et al., 2007; Fukuda, 2011), some of which possess Rab GAP activity. TBC1D9B is a GAP for Rab11, inactivating Rab11 by stimulating its GTP hydrolysis (Gallo et al., 2014). TBC1D9B is expressed in neurons and stimulates LMTK1 activity toward Rab11 during spine formation (Nishino et al., 2019). The mechanism by which LMTK1 regulates TBC1D9B remains to be elucidated. Nevertheless, by identifying TBC1D9B, the signaling cascade from Cdk5/p35 to Rab11 has been constructed (Figure 3).

## Physiological Functions and Pathological Roles of LMTK1

As mentioned above, LMTK1 negatively regulates endosomal trafficking in axons, dendrites, and spines. Therefore, a decrease in LMTK1 activity increases the supply of membrane components, leading to axon overgrowth, the stimulation of dendrite arborization, and an increase in the density of spines in cultured primary neurons. Not surprisingly, some abnormalities have been observed in the brains of LMTK1 KO mice. Although an overall malformation is not detected in the brains of adult LMTK1 KO mice, increased growth of apical and basal dendrites is observed in layer V neurons of the KO mouse at postnatal day 7 during development (Takano et al., 2014) and the number of synapses is increased in the cerebral cortex (Nishino et al., 2019). Interestingly, an increased spine density is linked to neurodevelopmental disorders, such as autism spectrum disorder (Hutsler and Zhang, 2010; Penzes et al., 2011; Yi et al., 2015; Yadav et al., 2017). The dysfunction of LMTK1 might contribute to the progression of developmental disorders, but unfortunately, the detailed analysis of behaviors has not been reported yet. We would like to introduce the cases of LMTK2 and LMTK3 here. LMTK2 KO mice do not show apparent abnormalities in brain structure and function, but exhibit defects in spermatogenesis at the stage of morphological differentiation from round to elongated spermatids (Kawa et al., 2006). LMTK3 KO mice show an increase in dopamine turnover (Inoue et al., 2014) and exhibit behavioral phenotypes associated with psychiatric diseases, cognitive dysfunction, and learning and memory impairments (Montrose et al., 2019).

Dysregulation of Rab11-dependent endosomal trafficking is reported in several neurodegenerative disorders, including AD, Parkinson’s disease, and Huntington’s disease (Kelly et al., 2012). In AD, Rab11 regulates the production of amyloid β through the endosomal trafficking of amyloid precursor protein (APP) and its cleavage enzymes, BACE1 and the presenilin complex (Udayar et al., 2013; Buggia-Prévot et al., 2014; Furusawa et al., 2019; Arbo et al., 2020). One of the recent research interests is the prion-like transportation of disease-related proteins, such as tau and α-synuclein, in the brain (Tarutani and Hasegawa, 2019). Rab11 plays a role in the exocytic release of α-synuclein in the exosome fraction (Chutna et al., 2014). Because LMTK1 is upstream of Rab11, LMTK1 itself is also plausibly involved in those diseases. The *aatk* gene has been identified as a haplotype-associated risk factor for early-onset frontotemporal dementia (FTD) in an Italian cohort (Ferrari et al., 2015), in which a single nucleotide polymorphism has implicated the downregulation of LMTK1. LMTK2 expression was recently reported to be decreased in patients with AD (Bencze et al., 2019; Mórotz et al., 2019). An examination of whether the downregulation of LMTK2 increases the trafficking of endosomes similarly to LMTK1 downregulation and vice versa would be interesting to analyze the expression and function of LMTK1 in the brains of patients with AD or animal models.

## Perspectives

In this review article, we describe a novel key role for LMTK1 in endosomal trafficking in neurons. Although more than 20 years have elapsed since LMTK1 was identified (Gaozza et al., 1997), it remains an enigmatic protein kinase. We recently reported the cellular functions of LMTK1 in modulating the formation of axons, dendrites, and spines through Rab11-dependent endosomal transport. Furthermore, we identified TBC1D9B Rab11 GAP as a direct target of LMTK1. However, many questions remain. The kinase activity of LMTK1 has not been confirmed. Therefore, it is not known how LMTK1 regulates the GAP activity of TBC1D9B. The downregulation of LMTK1 results in increased endosomal transportation, but its overexpression does not affect the basal endosomal transport. LMTK1 appears to play a role only in preventing overgrowth. If this function is validated, how is the basal transport regulated, and how does this mechanism differ from the mechanism suppressing overgrowth? What cargos are conveyed in LMTK1-targeted endosomes? LMTK1 KO or KD increased the anterograde movements of Rab11-positive endosomes, which may be driven by the kinesin motor protein. How does the LMTK1 signaling cascade regulate the movements of vesicles along MTs? Because the Rab11-dependent endosomal transport is associated with many physiological functions and neurological disorders, its regulator LMTK1 would also likely be involved in these processes. Thus, although LMTK1 is an intriguing neuronal modulator of endosomal trafficking, only a limited amount of information is available. Many open questions regarding LMTK1 remain to be explored.

## Author Contributions

SH, RW, AH, and MT wrote the text.

## Conflict of Interest

The authors declare that the research was conducted in the absence of any commercial or financial relationships that could be construed as a potential conflict of interest.

## Abbreviations

AF: actin filament
AD: Alzheimer’s disease
Cdk5: cyclin-dependent kinase 5
FTD: frontotemporal dementia
KD: knockdown
KN: kinase negative
KO: knockout
LMTK1: lemur tail kinase 1
PD: Parkinson’s disease
PP1: protein phosphatase 1
SNP: single nucleotide polymorphism
WT: wild type.
